# AlexandrusPS: A User-Friendly Pipeline for the Automated Detection of Orthologous Gene Clusters and Subsequent Positive Selection Analysis

**DOI:** 10.1093/gbe/evad187

**Published:** 2023-10-13

**Authors:** Alejandro Ceron-Noriega, Vivien A C Schoonenberg, Falk Butter, Michal Levin

**Affiliations:** Institute of Molecular Biology (IMB), Quantitative Proteomics, Mainz, Germany; Institute of Human Genetics, University Medical Center of the Johannes Gutenberg University Mainz, Department of Human Genetics, Mainz, Germany; Institute of Molecular Biology (IMB), Quantitative Proteomics, Mainz, Germany; Present address: Division of Hematology/Oncology, Boston Children's Hospital, Harvard Medical School, Boston, Massachusetts, USA.; Present address: Molecular Pathology Unit and Center for Cancer Research, Massachusetts General Hospital, Department of Pathology, Harvard Medical School, Boston, Massachusetts, USA.; Institute of Molecular Biology (IMB), Quantitative Proteomics, Mainz, Germany; Institute of Molecular Virology and Cell Biology, Friedrich-Loeffler-Institute, Greifswald, Germany; Institute of Molecular Biology (IMB), Quantitative Proteomics, Mainz, Germany

**Keywords:** AlexandrusPS, pipeline, positive selection

## Abstract

The detection of adaptive selection in a system approach considering all protein-coding genes allows for the identification of mechanisms and pathways that enabled adaptation to different environments. Currently, available programs for the estimation of positive selection signals can be divided into two groups. They are either easy to apply but can analyze only one gene family at a time, restricting system analysis; or they can handle larger cohorts of gene families, but require considerable prerequisite data such as orthology associations, codon alignments, phylogenetic trees, and proper configuration files. All these steps require extensive computational expertise, restricting this endeavor to specialists. Here, we introduce AlexandrusPS, a high-throughput pipeline that overcomes technical challenges when conducting transcriptome-wide positive selection analyses on large sets of nucleotide and protein sequences. The pipeline streamlines 1) the execution of an accurate orthology prediction as a precondition for positive selection analysis, 2) preparing and organizing configuration files for CodeML, 3) performing positive selection analysis using CodeML, and 4) generating an output that is easy to interpret, including all maximum likelihood and log-likelihood test results. The only input needed from the user is the CDS and peptide FASTA files of proteins of interest. The pipeline is provided in a Docker image, requiring no program or module installation, enabling the application of the pipeline in any computing environment. AlexandrusPS and its documentation are available via GitHub (https://github.com/alejocn5/AlexandrusPS).

**Significance**

Understanding the mechanisms and pathways that enable adaptation to different environments is crucial in evolutionary biology. However, existing tools for detecting such adaptive processes in protein sequences have limitations in terms of the computational complexity and required resources. AlexandrusPS is a user-friendly containerized pipeline that streamlines positive selection analysis of protein-coding genes on a genome scale by automating key steps, providing an easily interpretable output and facilitating high-throughput analyses on a desktop computer.

## Introduction

The evolution of protein sequences is influenced by the constraint of changes (purifying selection) or by the fixation of alleles that confer fitness advantage (positive selection; Maldonado et al. 2016). An essential metric to detect the selection type driving such sequence evolution is the nucleic acid and amino acid substitution rate, namely, the nonsynonymous (*d*_N_) to synonymous (*d*_S_) substitution rate ratio (ω = *d*_N_/*d*_S_). This measure has proven to be useful for understanding different evolutionary processes in comparative genomics (Clark et al. 2003; Chuang and Li 2004; Felsenstein and Felenstein 2004; Stark et al. 2007; Egan et al. 2008; Fedorova et al. 2008; Sánchez et al. 2011; Pan et al. 2013; Parker et al. 2013; Li et al. 2014; Bast et al. 2018; Glover et al. 2019; Liu et al. 2019; Forni et al. 2021; Policarpo et al. 2021). Such evolutionary analyses have profited from massive amounts of data derived from next-generation sequencing technologies, making comparative genomics analyses more attainable.

The enormous quantity of such data provides a valuable resource for researchers, but as the number of genomes continues to grow, downstream analyses have become increasingly challenging in terms of the quality and amount of data that need to be processed. This problem has led to the need for the development of specialized, efficient, and user-friendly bioinformatic tools that can help researchers with downstream tasks (Koepfli et al. 2015).

One of the most popular bioinformatic tools for applying maximum likelihood (ML) based models in evolutionary research to test the ratio between nonsynonymous and synonymous substitutions (ω = *d*_N_/*d*_S_) for multiple orthologous protein-coding sequences is CodeML (Yang 2007). CodeML is implemented in the Phylogenetic Analysis by Maximum Likelihood (PAML) program package (Yang 2007; Maldonado et al. 2016). Although the program is statistically robust and highly accurate in examining selective pressure (Yang et al. 2009; Zhai et al. 2012; Gharib and Robinson-Rechavi 2013; Macías et al. 2020), CodeML also faces limitations: 1) Being executed on a single processing unit renders operations on large sets of sequences highly time-consuming, driving the need for accessibility to high-performance computers (HPCs). 2) Each individual orthology group analysis needs to be separately prepared and executed by the user. 3) The execution requires a preceding accurate orthology analysis, which itself is challenging and can introduce errors to the analysis if not performed properly. 4) CodeML provides output that is difficult to interpret, especially for inexperienced users (Maldonado et al. 2013, 2016; Steffen et al. 2022).

To support less experienced users and minimize the manual operation of CodeML, several programs have emerged: JCoDA (Steinway et al. 2010), Armadillo (Lord et al. 2012), PAMLX (Xu and Yang 2013), IMPACT_S (Maldonado et al. 2014), PSP (Su et al. 2013), PhyleasProg (Busset et al. 2011), and Selecton (Stern et al. 2007). These programs use graphical interfaces or web-server implementations for single-gene family analysis. However, they are not suitable for the streamlined operation of CodeML for multiple analyses. Some additional softwares to solve these large-scale analysis challenges include VESPA (Webb et al. 2017), IDEA (Egan et al. 2008), and POTION (Hongo et al. 2015). These programs still have certain shortcomings: 1) The installation is complex. 2) They depend on large computational infrastructure such as HPCs. iii) They require advanced programming skills from the user. supplementary Table S1, Supplementary Material online provides a comprehensive comparison of the features and implementation properties of different available tools.

Here, we introduce AlexandrusPS, a high-throughput user-friendly pipeline designed to simplify the automated operation of established CodeML protocols. Containerized in a Docker image, AlexandrusPS was developed as a single command pipeline, minimizing user intervention in both installation and execution. The pipeline provides a well-organized output table, including all relevant results for drawing conclusions. All intermediate data, such as the results of the orthology analysis as well as multiple sequence alignments, are also retained. To enable full analysis flexibility for more experienced researchers, AlexandrusPS is an open-source software and thus enables modifications of parameters in all major configuration files.

## Implementation

### AlexandrusPS: Functionality

AlexandrusPS is a pipeline consisting of Perl and R scripts called by a main bash shell script and is available as a Docker image (https://github.com/alejocn5/AlexandrusPS). The only input needed from the user is FASTA files of CDS and amino acid sequences of all target proteins. AlexandrusPS leverages the ProteinOrtho program (Lechner et al. 2011) to discern and anticipate orthologous gene clusters (OGCs). These OGCs are selected for further investigation if they meet two criteria: First, they must encompass a minimum of three species; second, they exclusively consist of one-to-one orthologs, excluding any paralogs within the cluster spanning different species. The pipeline then utilizes PRANK to generate alignments and gene trees for each identified OGC. These gene trees are initially formatted in Nexus format but are subsequently converted to the dnd format, ensuring compatibility with subsequent analysis steps using CodeML.

To evaluate site-specific models (SSMs), the following model comparisons are performed: M0 versus M3, M1a versus M2a, and M7 versus M8. For branch models (BMs), ω values are estimated by evaluating M2 against a nearly neutral null model (M1a). For the branch-site model, M8a is compared with its null (M8a null), using a fixed ω assumption (ω = 1). Subsequently, Bayesian empirical Bayes analysis further identifies sites of positive selection, allowing posterior probability computation (Esteves et al. 2008).

These results are then used for likelihood ratio tests (LRTs) to determine whether the models reflect diversifying selection. For this, the log-likelihood score (2ΔlnL) between any two models is calculated. Subsequently, the *P*-value is determined by comparing each 2ΔlnL against the χ^2^ distribution using the respective degrees of freedom (DoF) for each model pair. Significant LRT results (FDR < 0.05) indicate a significant difference between the two models and thus imply an evolutionary explanation for these differences.

**Fig. 1.**
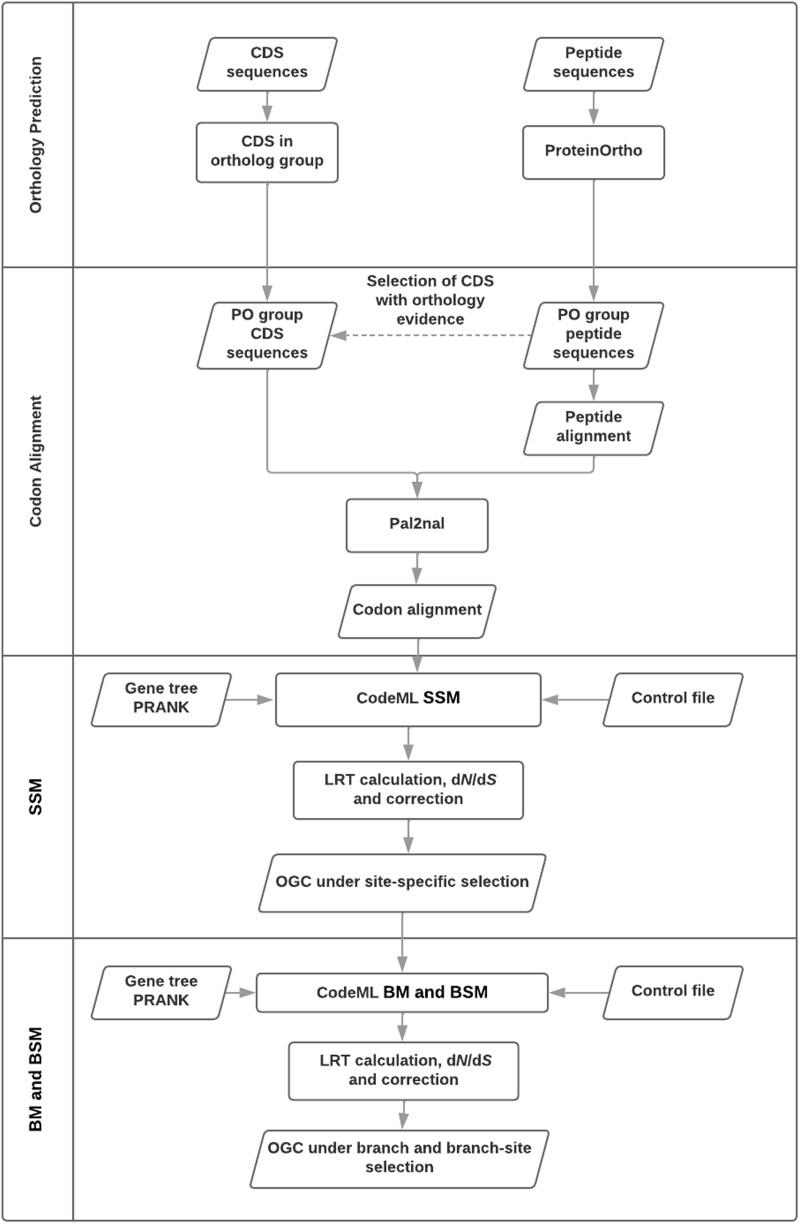
AlexandrusPS workflow. Flow chart describing the AlexandrusPS workflow, which sequentially combines four steps to finally execute CodeML and collect results. PO, ProteinOrtho; SSM, specific site model; BM, branch model; BSM, branch-site model; LRT, likelihood ratio test; OGC, orthologous gene cluster.

The main workflow of AlexandrusPS (fig. 1) is composed of four steps: 1) Orthology prediction by ProteinOrtho (Lechner et al. 2011); 2) multiple amino acid sequence alignment and gene tree generation by PRANK (Löytynoja 2014) and DNA codon sequence alignment by pal2nal (Suyama et al. 2006); 3) SSM calculations by CodeML (Yang 2007); and 4) branch and branch-SSM calculations by CodeML.

### AlexandrusPS: Input Files

#### FASTA Files of All Proteins of Interest

For each species included in the analysis, two FASTA files are needed: one with the amino acid and the other with the respective CDS sequences. Both files should contain the same number of sequences and their headers must be identical. AlexandrusPS can analyze all orthologous protein groups from protein-coding genes on a genome scale across multiple species. An example data set is provided with the pipeline to enable testing of the proper functionality of the pipeline (CDS and protein fasta files of this example data set are also included as supplementary Data 1, Supplementary Material online).

### AlexandrusPS: Output Files

#### Site-Specific Models

The CodeML output files are parsed into a CSV file. This file contains all OGCs organized in rows. Columns include OGC_ID, species included in the OGC, and ML results for all models with the respective metrics, such as likelihood (lnL), the number of parameters (np), ω (*d*_N_/*d*_S_), DoF, log-likelihood value (lnL), LRTs, and positively selected sites.

#### Branch and Branch-Site Models

The results of the LTR-based branch and branch-site model analyses (null model [H0] and alternative model [H1] of the branch-site test) for the OGC with significant signals of site-specific diversifying selection are written into final easily interpretable result files (the final output folder containing the result files of the example data set is included for illustration in supplementary Data 2, Supplementary Material online). We have introduced a significant improvement in comparison with other pipelines involving CodeML. AlexandrusPS employs a more refined selection procedure testing every individual branch. Specifically, within each OGC, each individual terminal branch is sequentially designated as the foreground, with all other branches considered as background. This approach, reminiscent of the methodology employed by Anisimova and Zhang in their study (Anisimova and Yang 2007), offers numerous advantages. Concentrating on a single foreground branch alongside multiple background branches, we constrain the calculations to the count of orthologs within the examined OGC, varying from a minimum of three to a maximum of all evaluated organisms. This unbiased choice of branches for foreground and background streamlines a more unbiased analysis, ultimately enhancing the comprehensiveness of our branch and branch-site model analysis.

### AlexandrusPS: Execution and Paralleling

Utilizing the inherent single-node architecture of CodeML, which operates on a single CPU, the parallelization process entails a series of systematic steps. Upon gathering the codon alignment, configuration files for the seven distinct CodeML models, and the phylogenetic tree specific to each OGC from prior stages, all relevant components are allocated to one of the accessible nodes, subsequently initializing the CodeML analysis. After extracting the values of likelihood (lnL), the number of parameters (np), ω (*d*_N_/*d*_S_), DoF, and log-likelihood value (lnL), the input and output files undergo compression. Subsequent to this, the node is freed to undertake the analysis of another OGC, continuing this iterative process until all OGCs have been analyzed. With this methodology, we enable increasingly efficient processing of large volumes of OGCs with augmenting amounts of available CPUs, making the pipeline optimally adjusted to run in HPC environments. We used AlexandrusPS for a positive selection analysis, including three of the nematode proteotranscriptomes (*Caenorhabditis elegans*, *Caenorhabditis briggsae*, and *Caenorhabditis inopinata*) established in Ceron-Noriega et al. (2023) on a tabletop PC with 20 CPUs and on an HPC system with 128 CPUs and could reduce computation time from 12.3 to 2.5 h, emphasizing the added value of using the pipeline on an HPC.

### Testing Positive Selection in Subgroups of the Phylogeny

AlexandrusPS enables positive selection analysis within OGC subgroups involving a minimum of three species, diverging from the conventional approach considering OGCs present in all species. This choice aims to address the potential impact of phylogenetic distances on positive selection signal dilution, which is often underestimated in large-scale analyses. Testing positive selection in subgroups relies on gene trees generated automatically by AlexandrusPS. Users should be aware that utilizing a gene tree can affect phylogenetic accuracy and positive selection detection due to distorted branch lengths, potentially leading to inaccurate substitution rate estimates and misidentification of positively selected genes. To validate positive selection signals, it is possible to confirm them with a reliable species phylogeny. After running AlexandrusPS, compressed intermediate data for each OGC can be accessed in the output folder. For validation with a species tree, replace the tree in the *.dnd.GenTree.nex file that is contained in the output/Results/<_ocgid_>.tar.gz/Orthology_Groups directory and run CodeML manually using the same config files that were already created (example config files are included in supplementary Data 3, Supplementary Material online).

### AlexandrusPS: Proof of principle

AlexandrusPS was successfully applied to perform a large-scale positive selection analysis using proteotranscriptomics data across 12 nematode species, including 77,000 protein sequences, resulting in 5,400 one-to-one orthologous groups, including orthologs from at least 3 species (Ceron-Noriega et al. 2023). This extensive phylogenetic analysis was executed on a tabletop PC with a processor of 8 cores/16 hyperthreads (8 Gb RAM each) and finished within 7 days. The analysis allowed interesting new insights into the evolutionary processes of this metazoan group and uncovered evolutionary events that suggest intriguing adaptive mechanisms. Notably, *Caenorhabditis japonica* exhibited an exceptionally high frequency of positive selection events. Interestingly, positively selected genes in *C. japonica* are closely linked to its distinctive phoretic lifestyle, setting it apart from other *Caenorhabditis* species, which are predominantly free-living. In stark contrast, *C. inopinata* displayed the lowest count of positively selected protein-coding genes. This stands in sharp contrast to the findings in its sister species, *C. elegans*, where we observed an enrichment of positively selected genes associated with muscle-related functions. This discrepancy is particularly striking given the close relationship between these two species and may be attributed to the long-term cultivation of *C. elegans* in laboratory conditions. The prevalence of muscle-related functions among the positively selected genes in *C. elegans* might reflect an adaptation to distinct demands for locomotion, such as moving on two-dimensional agar plates versus navigating a three-dimensional environment in soil or on decaying fruit. Additionally, we noted widespread adaptive evolution among ribosomal proteins in 7 out of the 12 species, highlighting that adaptation often occurs at fundamental gene regulatory levels rather than within highly specific functional subnetworks. Investigating these potent evolutionary changes is of significant interest and enhances our understanding of biological phenomena through in-depth phylogenetic comparisons among species that have more recently diverged.

## Conclusion

AlexandrusPS is a pipeline that is available in a Docker image to avoid the need for local installation of any modules or programs. It is provided as an open-source pipeline that allows the use of various CodeML models for molecular adaptive evolution (SSM, BM, and BSM) in parallel. It can run with default parameters, as it is based on standard protocols that allow the analysis of data sets that encompass protein-coding genes on a genome scale. Users are only required to provide the CDS and peptide FASTA files of the proteins of interest. With its simplicity of usage, AlexandrusPS offers distinct advantages over other programs.

AlexandrusPS automatically generates orthology relationships and identifies optimal orthology groups for positive selection analysis to avoid problems such as paralog introduction. It also generates a gene tree for each OGC and organizes, executes, and extracts all pertinent information from CodeML outputs. This completely automates the analysis with no need for intervention by the user. AlexandrusPS generates four main outputs: orthology relationships, site-specific positive selection results, branch and branch-site positive selection results, along with all intermediate files for each OGC. These intermediate files enable manual repetition of certain analyses for any individual OGC without having to repeat the entire process. AlexandrusPS allows users to run CodeML protocols on HPC systems but also on a desktop computer in an automated parallel manner.

We successfully applied AlexandrusPS to protein-coding genes on a genome scale to investigate positive selection in a phylogeny of 12 nematode species and obtained highly interesting results (Ceron-Noriega et al. 2023). We believe that this implementation will empower many more researchers to explore positive selection in any species of interest.

## Supplementary Material

evad187_Supplementary_Data

## Data Availability

There is no new data associated with this article. A detailed manual of the pipeline, all underlying scripts, and the Docker image can be found on the AlexandrusPS GitHub page (https://github.com/alejocn5/AlexandrusPS).
