# Supplementary material for: AlexandrusPS: A User-Friendly Pipeline for the Automated Detection of Orthologous Gene Clusters and Subsequent Positive Selection Analysis
Source: Genome Biol Evol. 2023 Oct 13;15(10):evad187. doi: 10.1093/gbe/evad187 (PMC10612477; doi:10.1093/gbe/evad187)
Supplement: evad187_Supplementary_Data [file evad187_supplementary_data.zip › Supplementary_Table_1.pdf]

Supplementary Table 1: Comparison of different positive selection software with AlexandrusPS

Program Name	AlexandrusPS	IDEA	Armadillo	IMPACT_S	JCoDA	Armadillo	PhyleasProg	POTION	PSP	Selecton	VESPA
Multi-task CodeML	YES	NO	NO	NO	NO	NO	NO	YES	NO	NO	YES
SM Analyses	YES	YES	YES	YES	YES	YES	YES	YES	YES	YES	YES
BM Analyses	YES	YES	YES	YES	NO	YES	YES	YES	YES	YES	NO
BSM Analyses	YES	NO	YES	YES	NO	YES	YES	YES	NO	YES	YES
Manual Installation	NO	YES	NO	NO	NO	NO	NO	NO	NO	NO	NO
Web-Server Implementation	NO	YES	YES	YES	NO	YES	YES	NO	YES	YES	NO
Config Files for CodeML produced automatically	YES	YES	YES	YES	YES	YES	NO	NO	YES	YES	YES
Orthology Relations for CodeML produced automatically	YES	NO	NO	NO	NO	NO	YES	YES	YES	NO	NO
MSA Files for CodeML produced automatically	YES	YES	YES	YES	YES	YES	YES	YES	YES	YES	YES
Phylogenetic Tree Files for CodeML produced automatically	YES	YES	YES	YES	YES	YES	YES	YES	YES	YES	YES
Automating Results Retrieval and LRTs	YES	YES	YES	YES	NO	YES	YES	YES	NO	NO	YES
HPC Applicability	YES	YES	YES	YES	NO	YES	YES	YES	YES	YES	YES
Open Source	YES	YES	YES	YES	YES	YES	YES	YES	YES	YES	NO
Parallelized Processes	YES	YES	YES	YES	NO	YES	YES	YES	YES	YES	YES
Docker Container	YES	NO	NO	NO	NO	NO	NO	NO	NO	NO	NO
