# Supplementary material for: AlexandrusPS: A User-Friendly Pipeline for the Automated Detection of Orthologous Gene Clusters and Subsequent Positive Selection Analysis
Source: Genome Biol Evol. 2023 Oct 13;15(10):evad187. doi: 10.1093/gbe/evad187 (PMC10612477; doi:10.1093/gbe/evad187)
Supplement: evad187_Supplementary_Data [file evad187_supplementary_data.zip › Supplementary_Data_2/Supplementary_Data_2/output/Orthology_Prediction/ProteinOrthoTable.proteinortho.html]

Proteinortho 6


### Proteinortho 6 : ProteinOrthoTable.proteinortho.tsv

×

### Extract fasta from selected group

---

Some text in the Modal..

Tip: You can click on the first 3 columns of a row (blue) to get information how to extract the genes/proteins from the given fastas.

The search for   
results in  groups.

manage

  
  
show IDs only

select

☑ Caljac.cur.pep.fasta☑ Chlsab.cur.pep.fasta☑ Gorgor.cur.pep.fasta☑ Homsap.cur.pep.fasta☑ Macmul.cur.pep.fasta☑ Nomleu.cur.pep.fasta☑ Pantro.cur.pep.fasta☑ Papanu.cur.pep.fasta☑ Ponabe.cur.pep.fasta

| # Species | Genes | Alg.-Conn. | Caljac.cur.pep.fasta | Chlsab.cur.pep.fasta | Gorgor.cur.pep.fasta | Homsap.cur.pep.fasta | Macmul.cur.pep.fasta | Nomleu.cur.pep.fasta | Pantro.cur.pep.fasta | Papanu.cur.pep.fasta | Ponabe.cur.pep.fasta |
| --- | --- | --- | --- | --- | --- | --- | --- | --- | --- | --- | --- |
| Loading data… |
| # Species | Genes | Alg.-Conn. | Calja | Chlsa | Gorgo | Homsa | Macmu | Nomle | Pantr | Papan | Ponab |
| --- | --- | --- | --- | --- | --- | --- | --- | --- | --- | --- | --- |

 
